# Assessment of Postvaccine Immunity against *Streptococcus pneumoniae* in Patients with Asplenia, including an Analysis of Its Impact on Bacterial Flora of the Upper Respiratory Tract and Incidence of Infections

**DOI:** 10.1155/2018/1691347

**Published:** 2018-12-31

**Authors:** Alina Olender, Katarzyna Małkińska, Jacek Roliński, Ewelina Grywalska, Elżbieta Pels, Jacek Tabarkiewicz

**Affiliations:** ^1^Department of Medical Microbiology, Medical University of Lublin, 20-093 Lublin, W. Chodźki 1, Poland; ^2^Chair and Department of Dermatology, Venerology and Paediatric Dermatology, Medical University of Lublin, 20-080 Lublin, Radziwiłłowska 13, Poland; ^3^Department of Clinical Immunology and Immunotherapy, Medical University of Lublin, 20-093 Lublin, W. Chodźki 4a, Poland; ^4^Chair and Department of Paedontics, Medical University of Lublin, 20-081 Lublin, Karmelicka 7, Poland; ^5^Centre for Innovative Research in Medical and Natural Sciences, Medical Faculty of the University of Rzeszow, ul. Warzywna 1A, 35-595 Rzeszow, Poland

## Abstract

*S. pneumoniae* is a microorganism that may cause a serious threat in postsplenectomy patients due to a potentially invasive course of infection. In order to assess a protective activity after vaccination with the 23-valent vaccine, we made an analysis of the level of antibodies in patients with asplenia compared to a control group of healthy donors. Additionally, colonization by potentially pathogenic microorganisms of the upper respiratory tract was analyzed to determine the carrier state by strains with vaccine serotype. No such strains were found in the research, yet three non-vaccine-serotype strains were found. Colonization of the upper respiratory tract by potentially pathogenic microorganisms may be connected with increased susceptibility observed and incidence of infections in patients with asplenia. However, colonization by *S. pneumoniae* may not have an effect on the level of specific antibodies with the 23-valent vaccine against *S. pneumoniae* (PPV23) in postsplenectomy patients and healthy people. The response to vaccination against *S. pneumoniae* showed a lower level of specific antibodies in patients with splenectomy performed more than 2 years before the test than in patients with a recently removed spleen, i.e., from 1 month to 2 years before the test. Vaccination against pneumococci also has positive effects on incidence of other etiology infections, which is of high significance in the prophylaxis of infectious diseases in this group of patients.

## 1. Introduction

Persons after spleen removal are a special group of patients in which a course of infection may be dangerous and life-threatening, especially in the case of encapsulated bacteria such as *Streptococcus pneumoniae*. Pneumococci infections in patients with asplenia cause a bigger threat of bacteremia and meningitis, in the form of invasive pneumococcal disease (IPD) or overwhelming postsplenectomy infection (OPSI), which is characterized by a high death rate mainly in such patients [1]. In this group of patients, as a result of the acquired immunodeficiency, a risk of cardiovascular complications is also higher due to the impaired coagulation processes, reduced blood filtration, and disorders of blood vessel endothelium [2]. After splenectomy, more than 50% of OPSI cases are caused by *S. pneumoniae*, which increases a risk of sepsis by 5-6 times [1]. Patients with upper respiratory infections and persons with colonization by this pathogen are a reservoir of pneumococci. *S. pneumoniae* is a microorganism that may come into the composition of physiological flora colonizing the upper respiratory tract. It usually occurs as asymptomatic carrier state, which in the event of immunodeficiency, may be a source of endogenic infections. It takes different forms depending on the age of patients. In the population of healthy children, the asymptomatic carrier state may occur within the range 20-60% (most often in children up to the 2nd year of life), whereas 5-30% in healthy adults, mainly above 65 years of age [3]. The possibility of simultaneous occurrence of numerous serotypes on the same area of the nasopharyngeal cavity and a differentiated duration of the carrier state are significant in colonization by *S. pneumoniae* [4]. Due to the type of the encapsulated serotype (there are over 90 types) strains of *S. pneumoniae* may differ in frequency of occurring and causing invasive infections [5]. Moreover, infections caused simultaneously by a few serotypes cannot be excluded. Colonization may be an important predisposition to endogenic infections especially in patients with disorders of the immunity system. Besides *S. pneumoniae*, other potentially pathogenic microorganisms that may colonize the upper respiratory tract are *Haemophilus influenzae*, *Neisseria meningitidis*, *Moraxella catarrhalis*, *Staphylococcus aureus*, and Gram-negative rods. Since *S. pneumoniae* is extremely dangerous for postsplenectomy patients, vaccination becomes an important action reducing the risk of dangerous infections. It is significant that prophylactic actions should also include research on their effectiveness concerning the occurrence of colonization as a potential source of endogenic infections and a general predisposition to infections that potentially may change nonspecifically as a result of vaccination.

The aim of this paper is an assessment of the immunologic response after vaccination with the 23-valent vaccine against *S. pneumoniae* of patients with asplenia towards a control group of healthy donors and an analysis of the correlation between a postvaccine response and colonization of the upper respiratory tract by *S. pneumoniae* and other pathogenic microorganisms, including a general incidence of infections in the examined group of patients.

## 2. Materials and Methods

### 2.1. Study Group

50 persons were enrolled into the study. 40 patients with asplenia—20 of which with a spleen removed for a period longer than 2 years before the test (study group A) and 20 persons with a recently removed spleen—i.e., from 1 month to 2 years (study group B). The study also included 10 healthy donors as a control group (group C). The examined persons were patients of the Immunology and Immunotherapy Clinic, Chair and Department of Clinical Immunology of the Medical University of Lublin. All persons gave informed consent to participate in the study, and the study was approved by the Bioethical Committee of Medical University of Lublin.

### 2.2. Microbiological Tests

The material to microbiological tests included nasal and pharyngeal swabs collected from patients before a planned vaccination against *S. pneumoniae* (0m-1st test) and 1-2 months after vaccination (Im-2nd test). The swabs were collected using a kit with a transport medium, and next a culture of microorganisms was performed on the following media: Columbia agar with 5% sheep blood, Mannitol salt agar, McConkey, Chocolate agar, and Sabouraud (bioMerieux, France), and the phenotypic identification with kits: the APINH, the APIStrep, the APIE, the APINE APIStaph, and the API AUX (bioMerieux, France). The numeric code of the identified strain was read out with the apiweb™ program (bioMerieux, France). The tests were conducted according to the routinely applied microbiological diagnostics.

### 2.3. Identification of the *S. Pneumoniae* Species and Its Serotypes

The phenotypically identified strains of *S. pneumoniae* were verified using the PCR method by detection of a gene encoding pneumolysin (gen *ply*) and autolysin (gen *lytA*) [6, 7].

All samples were submitted to a modified PCR reaction using 36 pairs of primers for the assayed serotypes or serogroups: 1, 2, 3, 4, 5, 6A/B/C/D, 7C, 7F, 8, 9A/V, 9N/L, 10A, 11A, 12A, 12F, 13, 14, 15A, 15B/C, 16F, 17F, 18C, 19A, 19F, 20, 22F, 23A, 23B, 23F, 24A/F, 31, 33F, 34, 35 B, 35F, and 38 [6–14]. Serotypes 6A and 6B were identified using sequential amplification by Pai et al. [11, 12].

While assaying serotypes, besides the PCR method, the Pneumotest-Latex kit (Statens Serum Institut, Denmark) was used as well [9, 11].

Doubtful isolates were submitted to further identification based on the sequencing of a fragment of the autolysin gene *lytA* [15]. The presence of a capsule was confirmed with the PCR method (*cps* gene) [9].

### 2.4. Assaying of the Level of Specific Antibodies in Serum

The tested material was peripheral blood collected from patients on an empty stomach in the morning hours from the basilic vein to standard vacutainers containing EDTA (Sarstedt, Germany).

The level of antibodies in class IgG against 23 encapsulated serotypes of *S. pneumoniae* with the highest pathogenic significance was assayed using the ELISA method with the ELIZEN Pneumococcus IgG test (ZenTech s.a., Belgium), which was performed according to the manufacturer's instruction.

We used blood serum in the test, collected from patients before vaccination (test 0s), 4 weeks after vaccination (test Is) and 12 weeks after vaccination (test IIs). The serum had been cryopreserved at a temperature of -70°C until the assays were performed. The relative change in antibody levels was calculated by subtraction of 0s concentration from Is or IIs concentrations and subtraction of Is concentration from IIs concentration.

### 2.5. Questionnaire Surveys

All persons included in the tests were given questionnaire surveys that included questions concerning frequency of infections they suffered, their duration, and occurrence before and after vaccination against *S. pneumoniae.*

### 2.6. Statistical Analysis

The test results were submitted to a statistical analysis, with calculation of frequency for quality variables and for quantity variables mean values ± standard deviation (SD) or median values; interquartile range (IQR) and minimum and maximum values were calculated. Because of non-Gaussian distribution of variables, statistical analysis of the verification of statistical hypotheses was based on nonparametric tests; the Kruskal-Wallis test was used to compare the groups, the Wilcoxon signed-rank test, and Friedman ANOVA were applied to compare the dependent variables. The analysis of differences in frequencies of characteristics was performed with the use of the *χ*^2^ test with Yates's correction and the Fisher's exact test. *p* < 0.05 was considered as statistically significant. The statistical analysis was made with the use of STATISTICA 10.0 software (StatSoft, Poland).

## 3. Results

The patients and healthy controls enrolled into the study were qualified to groups A, B, and C. Group A included 20 patients in which splenectomy had been made more than 2 years earlier. The average age of splenectomy was 28.7 ± 20.63. Group B included 20 patients, who underwent splenectomy less than 2 years before the study. The age of these patients amounted to 46.35 ± 16.61, and the control group C (10 healthy persons) has the average age of 34.68 ± 21.58. Among the patients with asplenia, there were 26 women and 14 men, whereas in the control group, 6 women and 4 men. The frequency of particular anatomical localizations of infections is summarized in Table 1. The antibiotics were used according to current recommendations of National Program of Use of Antibiotics 2016-2020 (Narodowy Program Ochrony Antybiotyków na lata 2016-2020) [16].

### 3.1. Characteristics of the Microbiological Flora of the Nose and Throat in the Examined Patients and Controls

Table 2 presents the results of nose and throat cultures received before vaccination (0m) and 1-3 months after vaccination (Im). To statistical purposes, particular groups of people were divided based on the number of detected pathogenic microorganisms. The group, in which potentially pathogenic species were isolated, included bacteria and fungi, which may colonize the respiratory tract and may also cause infections.

In most cases, the physiological flora from the throat included: *Streptococcus* group *viridans*, *Neisseria* spp., and *Haemophilus parainfluenzae.*

Microorganisms isolated from the nose in each of the tested groups were mainly methicillin susceptible *S. aureus* strains MSSA. In group A in the first test (0m) in three patients, in group B (0 m) in four persons, whereas in the test after vaccination in group A only in one person, in group B the number of colonized persons was the same as before vaccination. In healthy people from the control group (C), both before and after vaccination, *S. aureus* MSSA occurred only in one person. From the nose, we also isolated Gram-negative rods—*E. coli* and *S. marcescens* in group A in individual persons (0m), and in one person in the control group (0m)—*E. cloacae.*

Before vaccination, in the first throat culture (0m) in 22 patients, we found only the physiological flora. From the other patients, the physiological flora was isolated along with potentially pathogenic microorganisms. From 9 persons, *Staphylococcus aureus* (MSSA) was cultured. As a result of an extended genetic identification in three persons, presence of strains of *S. pneumoniae* was found, belonging to serotypes 6A (two isolates) and 15A (one isolate). In 5 patients bacteria genus *Enterococcus* and in 4 patients *Streptococcus pyogenes* were found. In 4 persons fungi *Candida albicans* and in one person *Candida glabrata* were also cultured. Moreover, *Streptococcus agalactiae*, *Pseudomonas aeruginosa*, *Aeromonas hydrophila*, *Streptococcus constellatus*, *Serratia marcescens*, and *Serratia odorifera* were cultured, one isolate each. *S. pneumoniae* in two cases occurred jointly with bacteria included in the physiological flora. They were serotypes 6A and 15A. In the case of one pneumococci of serotype 6A, besides the physiological flora, *S. pyogenes* was also present.

After vaccination, in the second throat culture (Im) in 41 persons, we only found the presence of the physiological flora. From the other 9 persons, besides the physiological flora, potentially pathogenic microorganisms were also isolated. In one person, *S. aureus* MSSA and *C. albicans* occurred simultaneously. In 4 patients *S. aureus* MSSA, in 2 persons *C. albicans*, from one patient *S. pneumoniae* of serotype 6A, and in one *S. pyogenes* were isolated.

### 3.2. The Level of Antibodies against *S. Pneumoniae*

A postvaccination response against pneumococci was analyzed in the group of patients after splenectomy and the control group of healthy people. The positive reaction to the vaccination against pneumococci was assumed as more than a double increase of antibody titers by comparing the values before vaccination (test 0s) and in Is assay after vaccination and before vaccination (test 0s) and in IIs assay after vaccination. The characteristics of the examined group and the analysis of the number of persons with a required increase of the titers of number of infections to vaccination, no changes in titers and decreased titers as well as incidence of infections are presented in Table 3.

Table 4 shows an analysis of a postvaccination response on different stages of vaccination. In group A (with a spleen removed more than 2 years before the test), there was a positive reaction to vaccination in 12 patients in the Is assay after vaccination and in 2 patients only in the IIs assay after vaccination. Lack of response to vaccination occurred in 8 patients in the Is assay after vaccination, and it remained in 6 patients in the IIs assay after vaccination. It was found in 2 patients that in the IIs assay after vaccination antibody titer dropped below the required double increase in spite of the fact that in the I assay a positive response to vaccination was found.

In group B (with a recently removed spleen from 1 month to 2 years), a positive reaction to vaccination was found in 13 patients in the Is assay after vaccination and in 2 patients only in the IIs assay after vaccination. Lack of response to vaccination occurred in 7 patients in the Is assay after vaccination, and it remained in 5 patients in the IIs assay after vaccination. It was found in 2 patients that in the IIs assay after vaccination the antibody concentration dropped below the required double increase in spite of the fact that in the Is assay a positive response to vaccination was found. In the control group in all persons shown, a positive reaction to vaccination was observed. A higher level of antibodies remained in the IIs assay after vaccination. The persons that positively responded to vaccination had a lower concentration of antibodies before vaccination than the persons that did not respond to vaccination.

In the individuals from whom *S. pneumoniae* was isolated, no correlation was found of a higher level of antibodies with the colonization by pneumococci strains. Depending on the serotype of *S. pneumoniae* we found, the following response was observed to vaccination: serotype 6A, two patients with a low response to vaccination (< a double increase); serotype 15A, a positive response to vaccination (a double increase).

The statistical analysis of the results concerning the difference in the values of specific antibody concentration IgG against pneumococci in serum collected from patients in the 3rd month (the IIs assay) after vaccination and in the 1st month (the Is assay) after vaccination did not show statistically significant differences between particular groups of the tested patients and the control group (*p* = 0.0512).

**Figure 1:**
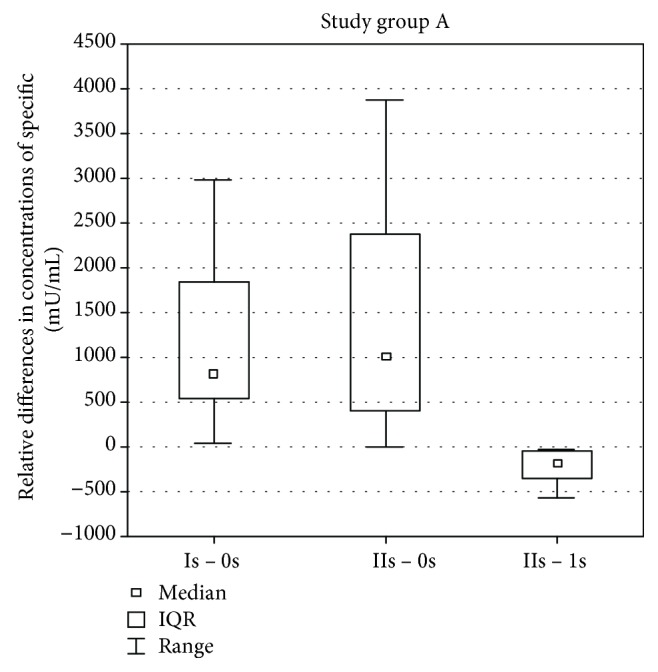
Differences between antibody concentration before vaccination 0s, 1 month after vaccination Is and 3 months after vaccination IIs of patients from group A.

While analyzing the results in the study group A (with a spleen removed more than 2 years before the test), a statistically significant difference was found in the difference of specific antibody concentrations against pneumococci by comparing the difference between the values in the Is assay after vaccination and before vaccination 0s and the values in the IIs and Is assays after vaccination (*p* = 0.0124). The difference was bigger while comparing the values in the Is assay after vaccination and before vaccination. A statistically significant change in the difference of specific antibody concentrations also occurred while comparing the values in the IIs assay after vaccination and before vaccination 0s and the values in the IIs and Is assays after vaccination (*p* = 0.0001). The difference was bigger while comparing the values in the II assay after vaccination and before vaccination 0s. No statistically significant change was found in the difference of antibody concentration by comparing the relative differences between the values in the Is assay after vaccination and before vaccination 0s and the values in the IIs assay after vaccination and before vaccination 0s (Figure 1).

**Figure 2:**
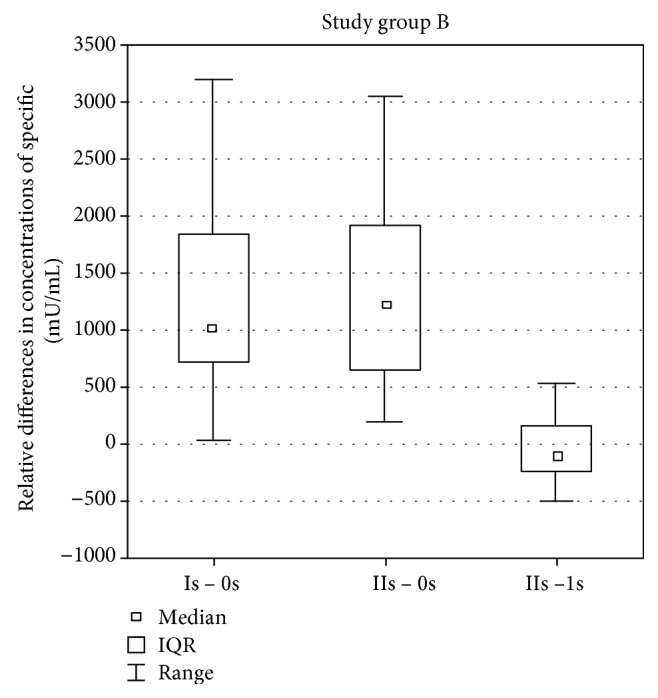
Differences between antibody concentration before vaccination 0s, 1 month after vaccination Is and 3 months after vaccination IIs of patients from group B.

While analyzing the results in the study group II (with a recently resected spleen from 1 month to 2 years), a statistically significant change was found in the difference of specific antibody concentration against pneumococci by comparing the difference between the values in the Is assay after vaccination and before vaccination 0s and the values in the IIs and Is assays after vaccination (*p* = 0.0017). The difference was higher while comparing the values in the Is assay after vaccination and before vaccination 0s. A statistically significant change in the difference of specific antibody concentrations also occurred while comparing the values in the IIs assay after vaccination and before vaccination 0s and the values in the IIs and Is assays after vaccination (*p* = 0.0001). The difference was bigger while comparing the values in the IIs assay after vaccination and before vaccination 0s. No statistically significant change was found in the difference of antibody concentrations by comparing the difference between the values in the Is assay after vaccination and before vaccination 0 s and the values in the IIs assay after vaccination and before vaccination 0s (Figure 2).

**Figure 3:**
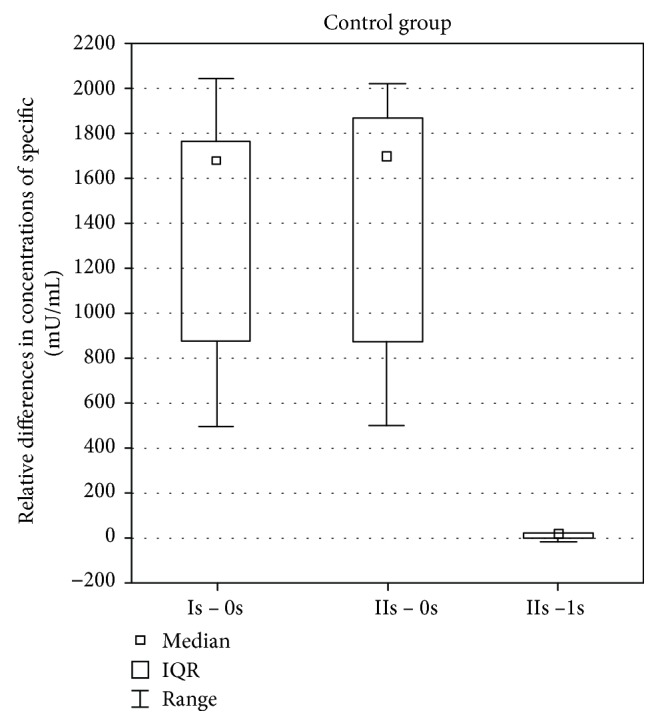
Differences between antibody concentration before vaccination 0s, 1 month after vaccination Is and 3 months after vaccination IIs in healthy controls.

While analyzing the results in the control group, a statistically significant change was found in the difference of specific antibody concentrations against pneumococci while comparing a difference between the values in the Is assay after vaccination and before vaccination 0s and the values in the IIs and Is assays after vaccination (*p* = 0.0051). The difference was bigger while comparing the values in the Is assay after vaccination and before vaccination 0s. A statistically significant change in the difference of specific antibody concentrations also occurred while comparing the values in the IIs assay after vaccination and before vaccination 0s and the values in the IIs and Is assays after vaccination (*p* = 0.0051). The difference was bigger while comparing the values in the IIs assay after vaccination and before vaccination 0s (Figure 3).

Based on the information received from the questionnaire surveys concerning infections in the tested patients, their characteristic properties and responses to vaccination against pneumococci was found that the group with a positive response to vaccination patients rarely underwent infections, and it referred to a shorter period from splenectomy in comparison to patients with lack of response to vaccination, who developed infections more often and where the period of asplenia was longer.

## 4. Discussion

Patients after a spleen resection may be more endangered, over time from the splenectomy, to colonization of the upper respiratory tract by potentially pathogenic microorganisms. It creates a possibility of endogenic infections, and the compromised immunity mechanisms of the local immunity also lead to a higher risk of exogenous infections. In the questionnaire surveys, more frequent infections were reported in the analyzed group of individuals with asplenia than in healthy controls. Vaccination against pneumococci, besides specific immunizing action, also showed stimulation of nonspecific immunity, which improved the situation of the patients. Similar effects were observed in persons with asplenia while using vaccines against flu, which decreased incidence of bacterial infections by *S. pneumoniae* and *H. influenzae* [17].

Prevaes et al. [18] in their research assessed the level of specific antibodies against *S. pneumoniae* in children that had been colonized earlier. Around half of the tested children were vaccinated with the 7-valent conjugate vaccine against pneumococci. The authors showed that the earlier colonization by pneumococci was connected with an increased concentration of specific antibodies IgG compared to a group of noncolonized children. However, specific antibodies did not prevent colonization by new strains of *S. pneumoniae.*

In our research, we observed that the antibody titer before vaccination was higher in the group that poorly responded to the applied vaccine. Similarly Chen et al., analyzing a postvaccination response in patients with chronic respiratory disorders, found that in the blood of patients poorly responding to vaccination the specific antibody titer was higher before vaccination than in persons properly responding to vaccination [19].

While analyzing the rate of increase of the level of antibodies after vaccination, it was found that in patients after splenectomy this increase was less dynamic in comparison to the control group. In patients with a resected spleen reacting positively to vaccinations, the increase of antibody concentration was about two times, whereas in the control group about six times higher.

Morgan and Tomich [20] drew the attention to a dangerous course of pneumococci infections in patients with asplenia and the need to diagnose OPSI quickly to implement an appropriate treatment, including antibacterial intravenous therapy, which may reduce mortality in patients with asplenia from 70% to 10-40%.

Colonization of the respiratory tract by nonvaccination serotypes of *S. pneumoniae* in our research did not have any impact on the level of postvaccination immunity. However, one cannot exclude the participation of *S. pneumoniae* strains in the potential threat of infection nor can this participation be overemphasized.

At the same time, Chironna et al. [21] described a case of mortal fulminate.ng pneumococci sepsis in a patient with asplenia, nonvaccinated against pneumococci. In the *post mortem* examination, they found a strain not included in the 23-valence vaccine. The authors emphasize that lethal infections may also threaten vaccinated persons with nonvaccination serotypes. The fact that there are patients after splenectomy, who were not vaccinated at all, indicates the need to make a register of patients with a high risk of development of pneumococci sepsis to provide them with protective vaccinations by the National Health System and antibiotic prophylactics as well as education [22, 23].

Unfortunately, most patients after splenectomy, who were obligatorily informed about a risk of sepsis and educated how to avoid this risk, do not follow the recommended guidelines [24].

Recommendations concerning procedures in patients after a spleen removal have been developed in the United Kingdom, Canada, USA, Australia, and New Zealand [25]. Davies et al. [17] noted that not in all patients a proper response is observed to vaccination against pneumococci with the 23-valent polysaccharide vaccine. The authors think that it may be caused by genetic factors, yet the tendency is especially discernible in the group of elderly persons and those submitted to splenectomy due to hematological reasons.

Recommendations concerning a repeated vaccination with a polysaccharide vaccine differ in different countries; in the United Kingdom, the guidelines recommend vaccination every 5-10 years and in Australia after 5 years. The need of repeated vaccination may be assessed based on specific antibodies titer. In relation to the above information, the authors recommend that patients, in whom a response to vaccinations was not found or was insufficient, shall be vaccinated with a conjugate vaccine in two doses [26].

Cherif et al. [26] in their research described the effectiveness of the 23-valent polysaccharide vaccine, which was used in patients submitted to splenectomy due to hematological reasons. A good response to vaccination was found in 72% of the examined patients. In the following years, they found episodes of pneumococcal infections only in the group with a poor postvaccination response. In this group of patients, revaccination did not lead to cause an increase of humoral immunity either. Patients with a poor postvaccination response were much older at the moment of vaccination than patients with an appropriate response to vaccination. In our research, a positive response to vaccination was found only in the case of one strain of serotype 6A and one belonging to serotype 15A. Turner et al. [27] in tests made in children to the 2nd year of age, concerning the carrier state of pneumococci in nasopharynx and the response to vaccination, found a positive response in the case of colonization by serotypes 19A, 23F, 14, and 19F. The authors state that the carrier status significantly increases the titer of IgG in serum. However, the relationship between the concentration of IgG and prevention or response to colonization of the nasopharynx by pneumococci is still difficult for interpretation.

Research conducted in the highly developed countries showed that reduction of colonization by pneumococci led to the occurrence of population immunity and reduction of the number of strains of *S. pneumoniae* resistant to antibiotics.

In our research, some patients were characterized by unstable concentration of antibodies after vaccination, which progressively slightly reduced, whereas in healthy controls such tendency does not. It may suggest the need of boost shots of vaccine to maintain the level of antibodies ensuring effective protection.

After vaccination with the 23-valent polysaccharide vaccine, the level of specific antibodies IgG significantly drops within 5 years. Yet the routine revaccination is not recommended due to a probability of occurrence of immunological hyperreactivity after the additional dose of vaccine. In the observed phenomenon of hyperreactivity, the first dose of the polysaccharide vaccine may cause a weaker reaction to the subsequent dose.

Kumar et al. [28] in 2003 in a double-blind controlled placebo study compared the effectiveness of two vaccines: polysaccharide and conjugate ones in adult recipients of a kidney transplant. However, they did not show statistically essential differences between the vaccines. In the research in 2007, Kumar et al. [29] described the differences in the preservation of a postvaccination response in dependence to the applied vaccine (polysaccharide or conjugate) in recipients of kidney after 3 years from the vaccination. The authors found a significant drop in the level of protective antibodies. The conjugate vaccine did not increase the durability of the postvaccination response.

## 5. Conclusion

Colonization of the upper respiratory tract by potentially pathogenic microorganisms may be connected with a higher susceptibility and frequency of infections observed in patents with asplenia. At the same time colonization by *S. pneumoniae* may not have an impact on the level of specific immunity after vaccination with the 23-valent vaccine against *S. pneumoniae* (PPV23) in patients after splenectomy and healthy persons.

The response to vaccination against *S. pneumoniae* in the tested patients after splenectomy was characterized by a lower level of specific antibodies in patients with splenectomy performed longer than 2 years before the test than in patients with a recently removed spleen, i.e., from 1 month to 2 years from the time of its removal. Vaccination against pneumococci also has a positive effect in terms of reduction of frequency of infections with other etiologies, which has an essential meaning in the prophylactics of infectious diseases in this group of patients. The authors suggested that vaccination against pneumococci shall be recommended to all patients after splenectomy without any medical contraindications. In case of patients who will undergo elective procedures of splenectomy, vaccination shall be considered before surgery. The efficacy of vaccination shall be monitored by measurement of specific antibody concentrations, and in individual cases of patients with poor response revaccination shall be considered.

## Figures and Tables

**Table 1:** The frequency of particular anatomical localizations of infections.

Infections	Group A (*n* = 20)	Group B (*n* = 20)	Group C (*n* = 10)	Total (*n* = 50)
Streptococcal pharyngitis	4 (20.00%)	2 (10.00%)	3 (30.00%)	9 (18.00%)
Upper respiratory tract	11 (55.00%)	11 (55.00%)	8 (80.00%)	30 (60.00%)
Lower respiratory tract	2 (10.00%)	3 (15.00%)	5 (50.00%)	10 (20.00%)
Urinary tract	1 (5.00%)	1 (5.00%)	1 (10.00%)	3 (6.00%)
Sepsis	1 (5.00%)	0 (0.00%)	0 (0.00%)	1 (2.00%)
Osteomyelitis	0 (0.00%)	1 (5.00%)	0 (0.00%)	1 (2.00%)

**Table 2:** Analysis of the microbiological flora isolated from the upper respiratory tract (nose and pharynx) before the vaccination planned (0m) and 1–2 months after vaccination (Im).

	The group A (*n* = 20/100%)		The group B (*n* = 20/100%)		The group C (*n* = 10/100%)	
	The nose					
The species	0m	Im	0m	Im	0m	Im
*S. aureus*	3/15%	1/5%	4/20%	4/20%	1/10%	1/10%
*E. coli*	1/5%	—	—	—	—	—
*S. marcescens*	1/5%	—	—	—	—	—
*E. cloacae*	—	—	—	—	1/10%	—
Sum	5/25%	1/5%	4/20%	4/20%	2/20%	1/10%

	The pharynx					
*S. aureus*	3/15%	2/10%	4/20%	2/10%	2/20%	—
*S. pneumoniae*	1/5%	1/5%	2/10%			
*S. pyogenes*	3/15%	—	1/5%	—	—	1/10%
*S. agalactiae*	1/5%	—	—	—	—	—
*S. constellatus*	—	—	1/5%	—	—	—
*Enterococcus spp.*	3/15%	—	2/10%	—	—	—
*S. marcescens*	1/5%	—		—	—	—
*S. odorifera*	—	—	1/5%	—	—	—
*P. aeruginosa*	1/5%	—	—	—	—	—
*A. hydrophila*	1/5%	—	—	—	—	—
*C. albicans*	1/5%	1/5%	2/10%	2/10%	1/10%	—
*C. glabrata*	—	—	1/5%	—	—	—
Sum	15/75%	4/20%	14/70%	4/20%	3/30%	1/10%

**Table 3:** Characteristics of the group of tested postsplenectomy patients (*n* = 40) in relation to the reaction to vaccination and the occurring bacterial infections.

		The reaction to vaccination		
		Increased titers (*n* = 25/62.5% all with asplenia)	No changes in titers (*n* = 11/27.5% all with asplenia)	Decreased titers (*n* = 4/100% all with asplenia)
Sex	Women	14/56%	8/72.7%	4/100%
	Men	11/44%	3/27.3%	—

Age (years)	Average ± SD	42.8 ± 18.8	36.8 ± 16.2	59.2 ± 10.2
	Median	44	26	61,5

Time from splenectomy (years)	Average ± SD	5.3 ± 7.1	6.4 ± 6.8	7.2 ± 7.1
	Median	2	4	5

The age at which the splenectomy was performed	Average ± SD	38.1 ± 20.4	30.7 ± 20.9	52.7 ± 16.6
	Median	41	18	53

Infections	Frequent >1 per month	5/20%	7/63.6%	3/75%
	Rare ≤1 per month	20/80%	4/36.4%	1/25%

**Table 4:** A response to vaccination against pneumococci in group A, group B, and control group C, including a positive response after vaccination or lack of response.

		Group A (*n* = 20)	Group B (*n* = 20)	Control group C (*n* = 10)
The reaction to vaccination—positive	Is a postvaccination study	12 (60%)	13 (65%)	10 (100%)
	IIs a postvaccination study	2 (10%)	2 (10%)	0 (0%)
	Together	14 (70%)	15 (75%)	10 (100%)

The reaction to vaccination—lack	Is a postvaccination study	8 (40%)	7 (35%)	0 (0%)
	IIs a postvaccination study	6 (30%)	5 (25%)	0 (0%)

The decrease in antibody concentration in this IIs study after vaccination despite a positive reaction in the study		2 (10%)	2 (10%)	0 (0%)

## Data Availability

The statistically analyzed data used to support the findings of this study are included within the article. The raw data used to support the findings of this study are available from Alina Olender, the first author, upon request.
